# Associations of PTPN22 and PADI4 polymorphisms with rheumatoid arthritis in ASWAN

**DOI:** 10.1186/s41927-025-00566-z

**Published:** 2025-09-22

**Authors:** Khaled A. A. Abdelgalil, Nihal Fathi, Fatma H. El Nouby, Nour A. Mohammed, Loay I. Aglan

**Affiliations:** 1 Department of Rheumatology, Rehabilitation & Physical Medicine, Faculty of Medicine, Aswan University, Aswan, Egypt; 2 Department of Rheumatology, Rehabilitation & Physical Medicine, Faculty of Medicine, Assiut University, Assiut, Egypt; 3 Department of Clinical Pathology, Faculty of Medicine, Aswan University, Aswan, Egypt

**Keywords:** Rheumatoid, PTPN22, PAD4, African, gene polymorphism

## Abstract

**Background:**

Rheumatoid Arthritis (RA) is a prevalent autoimmune disease affecting approximately 84,338 individuals in Egypt. Genetic predispositions, such as the PTPN22 and PADI4 genes, are linked to RA risk. PTPN22, a protein tyrosine phosphatase, a regulator of T-cell receptor signalling and PADI4, which is involved in citrullination, have shown varying levels of association with RA across populations. Studies have been inconclusive regarding their roles in RA susceptibility, progression, and activity.

**Patients and methods:**

A total of 240 participants were included in this study, RA patients and healthy controls from Aswan, Egypt. Genomic analysis was conducted to evaluate PTPN22 and PADI4 polymorphisms and their associations with RF and ACPA, Also, their correlation with disease activity markers, such as ESR, CRP, PGA and the Disease Activity Score (DAS28).

**Results:**

No significant association between PTPN22 and RA with p value ≥ 0.05 with good matching regarding age and sex. However, PTPN22 significantly correlated with RF and ACPA, with p-value of 0.006 and < 0.001, respectively, suggesting its diagnostic value in RA. No significant associations were found between PTPN22 and disease activity markers such as the ESR and CRP. In contrast, PADI4 levels were elevated in the control groups with p value < 0.001 which is against the study hypothesis, which conflicts with findings from other studies. Despite this, PADI4 demonstrated greater specificity (73.3%), in RA diagnosis than did PTPN22 (45.3%), making it a potential diagnostic marker in combination with RF and ACPA.

**Conclusion:**

Our study revealed no significant associations between PTPN22 or PADI4 polymorphisms and RA susceptibility in the Aswan population. However, both genes were correlated with diagnostic markers such as RF and ACPA. The PADI4 has potential as a diagnostic marker with high specificity.

## Introduction

Rheumatoid arthritis (RA) is among the most common rheumatic disorders with a total of 84,338 cases in 2019 in Egypt [1].

The exact cause of rheumatoid arthritis (RA) is not fully understood, but genetic and environmental factors play a role. The main genetic risk factor is the human leukocyte antigen (HLA), especially the HLA-DRB1 gene [2]. A second, more modest, association has been identified for the protein tyrosine phosphatase non receptor 22 (PTPN22) gene (SNP: rs2476601) [3]. Several studies have focused on the associations of the PTPN22 variant with RA risk and its clinical features, especially in the European population. The PTPN22R620W allele is associated with seropositive diseases [4, 5], anti-citrullinated protein antibodies (ACPAs) [6, 7], erosive diseases [8], and earlier disease onset [9]. In a stratified meta-analysis, PTPN22C1858T was more common in RF-positive patients than in RF-negative patients and was also more common in patients with anti-CCP antibodies than in those without [10].

Another important genetic variant, the peptidyl arginine deiminase 4 (PADI4) gene, is a member of the PADI family encoding enzymes involved in the posttranslational conversion of arginine within peptides to citrulline, calcium ion binding, and hydrolase activity, which play a role in hemostasis of the epidermis. PAD4 is expressed mainly in the hematological system and is believed to be involved in RA pathogenesis [11]. The strongest association was observed for an SNP (rs2240340) located in intron 3 of the PADI4_94 gene, which is associated with RA susceptibility and anticitrullinated peptide antibody (ACPA) positivity [12]. The association of this variant with RA susceptibility has been observed in different Asian populations [13, 14]. However, controversial results have been obtained in European Caucasian populations [15–17].

Rheumatoid arthritis (RA) susceptibility is influenced by genetic polymorphisms such as PTPN22 and PADI4, though their associations vary significantly across ethnic populations. The PTPN22 1858 C/T polymorphism has been robustly linked to RA in Caucasian populations, particularly in Northern Europe, but not in Asian cohorts, suggesting ethnic-specific genetic effects [18, 19].

Meta-analyses further highlight regional disparities, with stronger PTPN22 associations observed in Northern European populations compared to Mediterranean regions [20]. Similarly, PADI4 polymorphisms exhibit population-specific effects: while a functional PADI4 haplotype is associated with RA in Japanese populations, this association is absent in UK Caucasians [15, 16]. Despite these insights, conflicting results persist in Mediterranean and Middle Eastern populations, underscoring the need to evaluate these loci in understudied regions.

There is limited data concerning the association of PTPN22 and PADI4 polymorphisms with RA in Upper Egypt. This study investigates PTPN22 and PADI4 polymorphisms in the ASWAN population to address gaps in understanding RA genetic architecture in ethnically diverse cohorts and to assess their influence on the clinical and immunological features of the disease in Aswan, Egypt.

## Methods

### Study design and participants

We conducted a case‒control study at Aswan University Hospital. A total of 150 RA patients were enrolled in our study as case groups, and 90 healthy participants in the control group were matched for age, sex, and geographical origin with case subjects.

The inclusion criteria for RA patients were as follows: patients with rheumatoid arthritis who fulfilled the diagnostic criteria (at least four of the seven criteria for the diagnostic classification of RA established by the American College of Rheumatology (ACR 1987) and 6 of the 10 criteria of the 2010 ACR/ EULAR classification criteria of RA), as well as patients above 18 years of age.

The exclusion criteria for RA patients were as follows: patients with autoimmune diseases or arthritis other than rheumatoid arthritis; patients with chronic disease, alcoholism, liver disease, or kidney disease; patients under 18 years of age.

### Patients and methods

All patients underwent a comprehensive medical history review, clinical evaluation, and a thorough investigation. Disease activity was evaluated using the 28-joint Disease Activity Score (DAS28), and functional disability was assessed by the Health Assessment Questionnaire (HAQ). Inflammatory activity was evaluated via the erythrocyte sedimentation ratio (ESR) and C-reactive protein (CRP) level. Regarding Immunological analysis, all patients’ sera were tested for the presence of anti-CCP and RF.

### Determination of serum PTPN22and PADI4

We used a Sandwich ELISA kit from BT Lab to measure PTPN22 and PADI4 in serum samples from rheumatoid arthritis patients and healthy individuals. Serum was extracted via salting-out, and plasma samples were collected with EDTA or heparin.

### Assay principle

The kits use a sandwich immunoassay with pre-coated antibodies. Samples bind to the antibodies, followed by a biotinylated antibody and HRP. A substrate solution was added, resulting in a colour change proportional to protein concentration, with absorbance measured at 450 nm.

For PTPN22 and PADI4 analysis, genomic DNA was extracted from the collected whole blood of RA patients and healthy subjects, via the standard salting-out extraction method. All the DNA was stored at -20 °C until testing. The samples were genotyped for PTPN22 and PADI4 variants via ELISA according to the manufacturer’s instructions.

### Statistical analysis

All the statistics were performed via IBM SPSS Statistics 27.0. Continuous data, such as age and weight, were presented as the means ± SDs, while data that are not continuous are shown as median (IQR).

To compare groups, the following statistical tests were used: - For parametric and continuous data, the Student t-test compares group means, and the Chi-square test assesses categorical variable associations.

- For non-parametric data, the Mann-Whitney U test evaluates differences, and Spearman’s rank correlation coefficient examines relationships between two variables.

Receiver Operating Characteristic (ROC) curve analysis identifies optimal cut-off values and computes sensitivity, specificity, and the Area Under the Curve (AUC).

## Results

### Participant characteristics

A total of 240 participants were enrolled in our study, of which 150 RA patients and 90 healthy participants composed the control group. In terms of sex, the majority of the participants in both groups were female. There were slightly more male participants in the control group (22.22%) than in the RA group (15.33%), but this difference was not significant (*p* = 0.178). The mean age of the RA patients was 44.55 years, whereas the mean age of the controls was 45.4 years (Table 1).

**Table 1:** Comparison of the characteristics of the two groups by sex and age

		Group		Test of Significance		
		Controls	Cases			
		*N* (%) Mean ± SD	*N* (%) Mean ± SD	Value	*p*-Value	Significance
Sex	Male	20 (22.22%)	23 (15.33%)	X² =1.815	0.178	NS
	Female	70 (77.78%)	127 (84.67%)			
Age		45.4 ± 13.29	44.55 ± 13.36	t = 0.476	0.634	NS

Chi-square test of significance (X^2^)

Student’s t test of significance (t)

Table (1) compares cases and controls by sex and age. Slightly more males were among the controls (22.22%) than the cases (15.33%), but the difference was not significant (*p* = 0.178). The mean ages of both groups were also similar, confirming good matching by age and sex.

### Characteristics of the RA patients and laboratory investigations

After the RA patients were evaluated, their history revealed that the mean duration of illness was 7.35 years, with a minimal duration of illness of 1 year and a maximal duration of 23 years. More than 80% of the patients had an irrelevant family history. Our data, revealed that rheumatoid factor (RF) was positive in 64% of the patients. the mean ESR was 53.09, with a minimum value of 10 and a maximum value of 105, whereas the median CRP was 6, ranging from 6 to 24. Moreover, the mean DAS28 ESR was 5.14, and the mean PGA was 6.41. In addition, 99 patients were positive for ACPAs (Table 2).

**Table 2:** Characteristics of the RA patients and laboratory investigations

		Mean ± SD/*N*	Median (IQR)/%	Min–Max
Family history	Irrelevant	123	82.0%	
	Positive	27	18.0%	
Duration of RA		7.35 ± 5.03	6 (3–10)	1–23
RF	Negative	54	36.0%	
	Positive	96	64.0%	
ESR		53.09 ± 20.57	50 (40–65)	10–105
CRP		15.16 ± 16.67	6 (6–24)	0–64
DAS28 ESR		5.14 ± 1.31	5.22 (4.35–6.04)	1.9–7.9
PGA		6.41 ± 2.04	7 (5–8)	2–10
ACPA	Negative	51	34.0%	
	Positive	99	66.0%	

Table (2) shows the characteristics of the RA cases. Approximately 18% of the rheumatic patients had a positive family history. The mean RA duration was 7.35 years (± 5.03). More than half of the patients (64.0%) had positive RFs. The mean ESR was 53.09 (± 20.57), with a median CRP of 6 (6–24). Moreover, the mean DAS28 ESR and PGA were 5.14 (± 1.31) and 6.41 (± 2.04), respectively. Approximately 66% of the patients were ACPA positive.

#### PTPN22 and PAD4

According to the Mann‒Whitney Test of Significance, there is no significant difference between the two groups regarding PTPN22 expression levels with p value ≥ 0.05, while there is a significant difference regarding PAD4 being higher in the control groups compared to RA cases group with p value < 0.001which is against the study hypothesis (Table 3).

**Table 3:** Comparison of PTPN22 & PAD4 expression between the two groups

	Group		Mann‒Whitney Test of Significance		
	Controls	Cases			
	Median (IQR)	Median (IQR)	Value	*p* Value	Significance
PTPN22	0.62 (0.55–0.72)	0.58 (0.49–0.72)	-1.575	0.115	NS
PAD4	0.62 (0.47–0.74)	0.46 (0.36–0.57)	-5.412	< 0.001	S

Table (3) shows a statistically significant difference between the two groups in terms of PAD4, which was greater in the control group (0.62) than in the case group (0.46), with a p value < 0.001. However, there were no significant differences between the cases and controls regarding PTPN22, with a p value ≥ 0.05.

- Correlations between PTPN22 characteristics and laboratory findings.

To assess the relationship between PTPN22 and RA patient characteristics, we used the Mann‒Whitney test of significance. Our data revealed that there was a significant difference between PTPN22 levels and sex, with a p-value of 0.036. RF and ACPA levels were significantly different from PTPN22 levels, with p-values of 0.006 and < 0.001, respectively. Instead, the data concerning family history were not significantly different from those concerning PTPN22, with a p value of 0.597 (Table 4).

**Table 4:** Correlations between PTPN22 characteristic

		PTPN22	Mann-Whitney Test of Significance		
			Value	p-Value	Significance
Sex	Male	0.72 (0.52–0.84)	-2.092	0.036	S
	Female	0.58 (0.48–0.69)			
Family history	Irrelevant	0.57 (0.49–0.72)	-0.528	0.597	NS
	Positive	0.6 (0.5–0.72)			
RF	Negative	0.54 (0.49–0.6)	-2.737	0.006	S
	Positive	0.63 (0.5–0.74)			
ACPA	Negative	0.52 (0.47–0.58)	-3.803	< 0.001	S
	Positive	0.62 (0.5–0.75)			

Table (4) shows that PTPN22 expression was significantly associated with sex and was lower in females, with a p-value of 0.036. Moreover, PTPN22 expression was greater in patients with positive RFs and positive ACPAs, indicating a statistically significant association between PTPN22, RF and ACPAs. However, there was no significant association with family history, with a p-value ≥ 0.05.

On the other hand, the correlation between PTPN22 levels and laboratory findings in the RA patient group determined via Spearman’s coefficient test revealed a positive significant correlation between PTPN22 levels and the DAS28 ESR, with a p-value = 0.011. However, our analysis revealed an insignificant relationship between PTPN22 levels and the other laboratory parameters, such as ESR, CRP, and PGA, with p values ≥ 0.05 (Table 5).

**Table 5:** Correlations among PTPN22 laboratory investigations

		Age	Duration of RA	ESR	CRP	DAS28 ESR	PGA
PTPN22	Spearman’s Coefficient	0.058	0.046	0.132	0.079	0.208^*^	0.136
	p Value	0.479	0.574	0.107	0.338	0.011	0.098
	Significance	NS	NS	NS	NS	S	NS

Table (5) shows the correlations among PTPN22, patient characteristics and the results of laboratory investigations. PTPN22 was significantly positively correlated with the DAS28 ESR (p value = 0.011). However, the other variables did not have a statistically significant p value (≥ 0.05).

b) Correlation between PAD4, characteristics and laboratory investigations.

According to the Mann‒Whitney test of significance in measuring the correlation between PAD4 and RA patient group characteristics, the analysis revealed that there was a significant difference between PAD4 levels and ACPA positive cases, with a p-value < 0.001; however, there was an insignificant difference between PAD4 and sex, family history and RF, with a p-value ≥ 0.05 (Table 6).

**Table 6:** Correlations between PAD4 characteristics

		PAD4	Mann‒Whitney Test of Significance		
			Value	p Value	Significance
Sex	Male	0.46 (0.38–0.53)	-0.378	0.705	NS
	Female	0.45 (0.36–0.59)			
family history	Irrelevant	0.45 (0.37–0.55)	-1.304	0.192	NS
	Positive	0.5 (0.34–0.74)			
RF	Negative	0.42 (0.35–0.54)	-1.693	0.090	NS
	Positive	0.47 (0.38–0.58)			
ACPA	Negative	0.4 (0.33–0.5)	-3.424	< 0.001	S
	Positive	0.47 (0.4–0.6)			

Table (6) shows that PAD4was more common in patients with positive ACPAs, with a p value < 0.001. However, PAD4 was not significantly associated with sex, family history or RF, with a p value ≥ 0.05.

Spearman’s coefficient test revealed a positive correlation between PAD4 and DAS28 ESR, with a p-value = 0.025, whereas the other variables were not correlated with PAD4, ESR, CRP or PGA, with a p value ≥ 0.05 (Table 7).

**Table 7:** Correlations between PAD4 laboratory investigations

		Age	Duration of RA	ESR	CRP	DAS28 ESR	PGA
PAD4	Spearman’s Coefficient	0.046	0.097	0.060	-0.009	0.183*	0.033
	p Value	0.579	0.23 6	0.466	0.909	0.025	0.686
	Significance	NS	NS	NS	NS	S	NS

Table (7) shows the correlations among PAD4, characteristics and the results of laboratory investigations. PTPN22 was significantly positively correlated with the DAS28 ESR (p value = 0.025). However, the other variables did not have a statistically significant p value (≥ 0.05).

#### Overall sensitivity and specificity of PTPN22 and PAD4

**Fig. 1:**
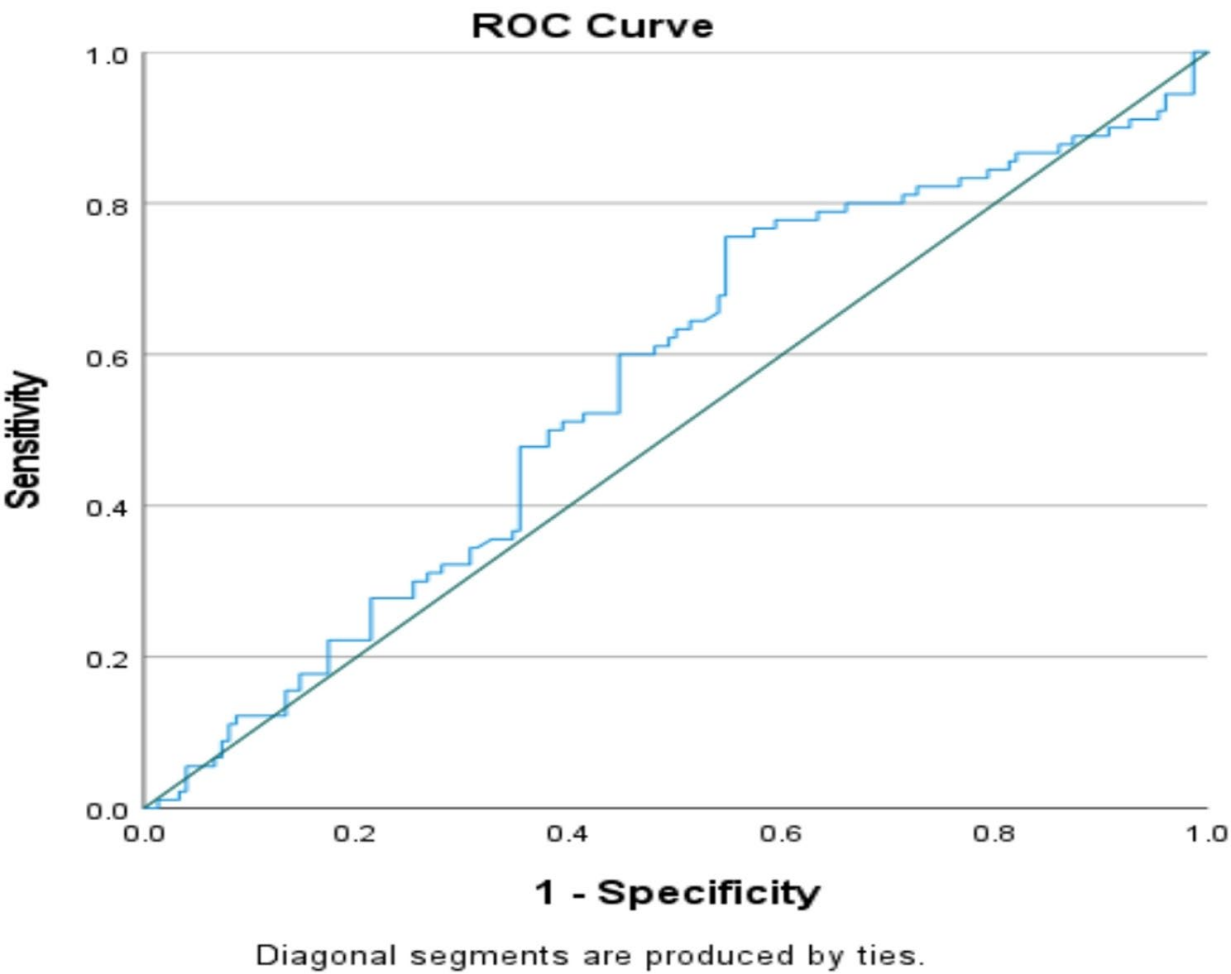
Serum PTPN22 ROC curve

**Fig. 2:**
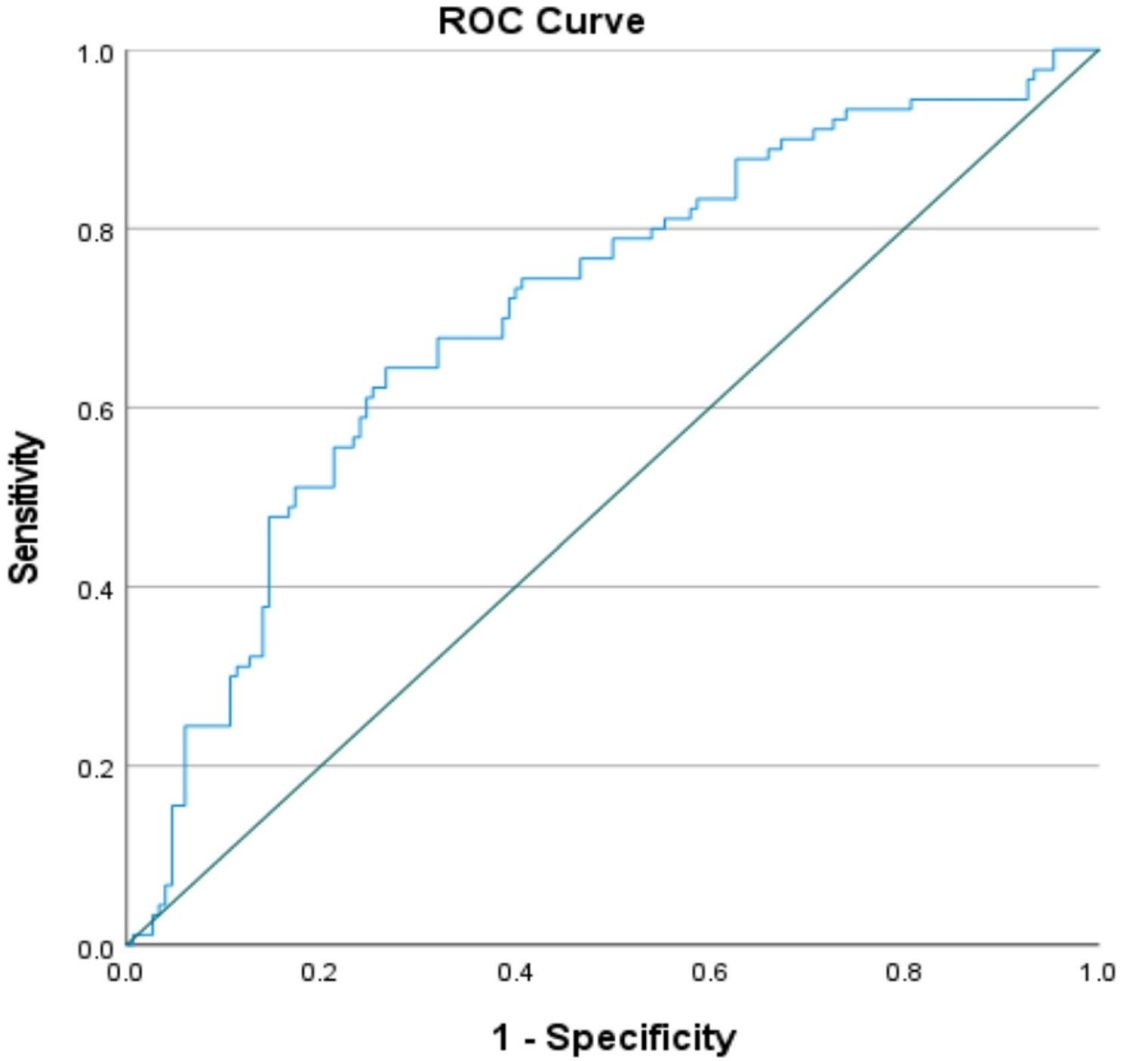
Serum PAD4 ROC curve

Receiver operating characteristic(ROC) curve analysis revealed that the overall sensitivities and specificities for the tested biomarkers (PTPN22 and PAD4 markers) were different. Compared with the PAD4 test, the PTPN22 test had better sensitivity than did the PAD4 test (75.6%, 64.6%). However, the PAD4 test had greater specificity (73.3%), making it a good negative test, whereas the specificity of the PTPN22 test was 45.3% (Figs. 1 and 2).

(Figs. 1 and 2): the overall sensitivities and specificities for the tested biomarkers (PTPN22 and PAD4 markers) were different. Compared with the PAD4 test, the PTPN22 test had better sensitivity than did the PAD4 test (75.6%, 64.6%). However, the PAD4 test had greater specificity (73.3%), making it a good negative test, whereas the specificity of the PTPN22 test was 45.3%.

The area under the curve (AUC), which represents the overall diagnostic performance of each marker, was 0.56 for PTPN22, with a cut-off value of 0.55, while the PADI4 AUC was 0.708, and the cut-off value was also 0.55, with higher AUC values indicating better diagnostic accuracy.

With respect to PTPN22, the results were not significant, as the P value was greater than 0.1, which indicates that there was no evidence against the null hypothesis; however, the PAD4 results were significant, with P-values less than 0.05 (Table 8).

**Table 8:** Overall sensitivity and specificity of PTPN22 & PAD4

Markers (Units)	PTPN22	PAD4
Cut-off value	0.553	0.554
AUC	0.56	0.708
S.E.M	0.038	0.034
P value	0.115	< 0.001
95% CI	0.485 to 0.635	0.64 to 0.776
Sensitivity %	75.6	64.4
Specificity %	45.3	73.3

Table (8) shows the PTPN22 test had better sensitivity than did the PAD4 test (75.6%, 64.6%). However, the PAD4 test had greater specificity (73.3%), making it a good negative test, whereas the specificity of the PTPN22 test was 45.3%.

## Discussion

This study aimed to investigate the associations of PTPN22 and PADI4 gene polymorphisms with rheumatoid arthritis (RA) susceptibility and disease activity in the Aswan population. Our results did not reveal a significant association between these genes and RA risk, but we observed correlations with disease activity and diagnostic markers, such as RF and ACPA. These findings are consistent with several studies but also highlight notable differences across populations.

### PTPN22 and RA susceptibility

Our study did not reveal significant differences in PTPN22 expression between RA patients and controls with p-value ≥ 0.05, which aligns with findings from a Turkish study that also revealed no association between PTPN22 gene polymorphisms and RA [18]. Additionally, a meta-analysis confirmed that the PTPN22 1858 C/T polymorphism is associated with RA in Caucasians but not in Asians [19]. However, studies in Mediterranean populations have reported conflicting results, with some showing a significant association in northern Europe and others, suggesting regional variability [20].

Numerous studies in Egypt highlight a correlation between the PTPN22 rs2476601 polymorphism and rheumatoid arthritis (RA) susceptibility.

- For example, the T allele of PTPN22 was significantly more prevalent in RA patients compared to controls (OR = 4.89–5.98), linking it to both susceptibility and functional impairment [21].
- A multi-center study also identified PTPN22 as a risk factor, particularly in subsets positive for anti-citrullinated protein antibodies (ACPA) [22].

However, our study in Aswan found no significant association, possibly due to regional genetic differences or small sample sizes. This aligns with an Algerian study that noted the T allele’s link to RF + RA subsets (OR = 8.53) but no overall correlation with RA susceptibility, highlighting the complexities of genetics in disease [23].

(see Tables 9 and 10: Comparison of PTPN22 and PADI4 gene polymorphisms in Egypt and across different regional and global populations)

### Diagnostic and prognostic value of PTPN22

Our data suggest that PTPN22 is significantly associated with RF and ACPA levels with p-value ≥ 0.006 and < 0.001, respectively, which supports its potential as a diagnostic marker, as noted in previous research [21–23]. However, its value in disease progression remains unclear. In contrast to our findings, Lie et al. (2007) demonstrated that PTPN22 is linked to the progression of joint damage in RA patients, suggesting that it could also play a role in disease severity and long-term outcomes [8].

### PADI4 and RA risk

The PADI4 expression levels were lower in our RA patients than in the controls, which contrasts with findings from studies in Iran, Swede, Japan and Egypt [24–27]and agreed with Algerian study [23].

Studies have shown that specific PADI4 SNPs are associated with RA, suggesting that differences in genetic architecture may explain the inconsistencies observed in different regions [15, 16]. This highlights the importance of considering geographic and ethnic diversity in genetic research on RA.

Genetic polymorphisms and their associations with diseases can vary by population. The PADI4_94 polymorphism (rs2240340) is strongly linked to rheumatoid arthritis (RA) in Asian populations but shows inconsistent results in Egypt, emphasizing the need for population-specific studies [28].

A study in Egypt investigated three PADI4 SNPs—PADI4-92, PADI4-96, and PADI4-102—using PCR-RFLP and found a significant association between PADI4-92 and PADI4-102 in RA cases, while PADI4-96 showed no significant association. This suggests varying expression levels of PADI4 based on specific SNPs related to RA [29].

Despite the lack of association between PADI4 and RA diagnosis in our study, PADI4 showed a significant correlation with ACPA positivity (*p* < 0.001), similar to [27, 30] studies, that demonstrated that PADI4 could serve as a diagnostic marker in RA.

### Sensitivity and specificity of PTPN22 and PADI4

Our analysis revealed significant diagnostic potential for PADI4, with a sensitivity of 64.4% and a specificity of 73.3%, indicating that it could be a reliable negative test for RA. In contrast, PTPN22 did not show significant diagnostic value in our study. These findings are consistent with those of [27], who reported similar results in Egyptian cohorts, confirming the diagnostic utility of the PADI4.

**Table 9:** (Summary comparison of PTPN22 and PAD4 gene polymorphisms in rheumatoid arthritis among individuals in Egyptian)

Aspect	PTPN22 in Egyptians	PADI4 in Egyptians
Susceptibility	Strong association (rs2476601) [21, 22]	Weak/no association [22]
Diagnostic Role	Linked to RF/ACPA positivity	High specificity (73.3%) with RF/ACPA
Population Trends	Consistent risk factor across studies	Variable, population-dependent associations

While PTPN22 is a robust genetic risk factor for RA in Egyptians, PADI4 polymorphisms show weaker or inconsistent associations. This divergence highlights the importance of regional genetic studies and suggests that PTPN22-targeted therapies (e.g., modulating T-cell pathways) may hold more promise for Egyptian RA patients compared to PADI4 inhibition.

(See Table 9 for a summary comparison of PTPN22 and PADI4 gene polymorphisms in Egyptian RA patients.)

- Key Comparisons of PTPN22 and PADI4 Across Populations: Our findings, set against the backdrop of global research, reveal that the roles of the PTPN22 and PADI4 genes in rheumatoid arthritis (RA) are greatly influenced by population differences (see Table 10). Data from Aswan show no general link between PTPN22 and RA, but a significant correlation exists with seropositive forms of the disease (RF⁺, ACPA⁺), aligning with other Arab cohorts. While PADI4 doesn’t appear to have a strong susceptibility role, it may serve as a valuable diagnostic tool when considered with RF and ACPA.

In contrast, studies in Europe and North America identify PTPN22 as a key susceptibility gene, particularly in anti-CCP-positive RA. Meanwhile, Asian populations demonstrate a solid association with PADI4, especially among smokers and carriers of the HLA-DRB1*04 allele. These differences illuminate the impact of ethnic and genetic backgrounds on RA pathogenesis, highlighting the need for region-specific genetic screening strategies.

**Table 10:** Comparison of PTPN22 and PAD4 gene polymorphisms in rheumatoid arthritis across aswan, Egyptian and Arab populations, Sub-Saharan africa, and globally

Population	Gene	Susceptibility Role	Clinical/Functional Role
Aswan, Egypt	PTPN22	No overall RA risk. Strongly linked to seropositive RA (RF + *p* = 0.006 /ACPA+; *p* < 0.001).	Correlates with higher RF/ ACPA disease activity and progression rather than susceptibility.
	PADI4	No significant RA association.	Enhances diagnostic specificity (73.3%) when combined with RF/ACPA.
Other Egyptian /Arab	PTPN22	o Strong association reported in Egyptian [21, 22]. Moderate association in RF + subsets (e.g., Algeria: OR = 8.53 for T allele). No significant association with overall RA susceptibility [23]	Limited data available [22]
	PADI4	o No confirmed RA association. [22] o Limited data [27]	Limited data Minimal impact on diagnosis or severity.
Sub -Saharan Africa	PTPN22	Rare allele (rs2476601); minimal impact on RA risk. [23]	Not studied.
	PADI4	No available data on RA susceptibility.	Understudied.
Europe	PTPN22	Strong RA risk (OR = 2.16–10.35), especially in anti-CCP + RA. [31]	Linked to Accelerated joint damage; male-predominant risk. [8]
	PADI4	Weak/no association (e.g., UK: OR = 1.01, *p* = 0.72) [15] lack of association with RA. [32]	Minimal role in RA pathophysiology. Marginal role in citrullination pathways. [16]
Asia	PTPN22	No rs2476601 link (rare). Promoter polymorphism -1123G > C linked to RA (OR = 1.5). [33]	May influence earlier disease onset.
	PADI4	Strongest RA association (OR = 12.19 in ACPA + Kazakhstani RA). [33]	Synergizes with HLA-DRB1*04 and smoking to drive ACPA + RA. [34]
North `America	PTPN22	rs2476601 confirmed as risk factor (OR = 1.49). [20]	Role in seropositive RA similar to Europeans.
	PADI4	Population-specific effects (e.g., rs2240340 linked in cohorts with mixed ancestry). [34]	Limited standalone diagnostic utility.

Table 10: illustrates the comparison of PTPN22 and PADI4 gene polymorphisms across different regional and global populations.

## Strength points and limitations

- This study has several strength points which can help in future research and and clinical applications:

1. Being in a specific in geographical and demographical parameters as it’s in Aswan population to evaluate their genetic architecture of 240 participants.
2. It investigates biomarkers beyond disease susceptibility, analyzing their correlation with RF, ACPA, and inflammatory markers, providing insights into the impact of PADI4 and PTPN22 on disease activity and prognosis.
3. The study contributes to personalized medicine by identifying genetic markers for RA susceptibility, potentially leading to tailored treatments for that population.
4. Results can be compared with other regional studies, enhancing understanding genetic influences on RA across diverse populations.

- Our study also has some limitations:

1. To address the limitations of the ELISA technique, consider using PCR to amplify DNA for direct identification of polymorphisms (SNPs). ELISA is not a substitute for PCR in directly genotyping PTPN22 or PADI4, but it is useful in rheumatoid arthritis (RA) research. It connects genetic variants to outcomes like autoantibody levels and allows for high-throughput screening in resource-limited settings. ELISA is more cost-effective for large sample sizes, providing insights beyond basic SNP detection. However, PCR is still the preferred method for accurate and scalable SNP detection.
2. A larger sample size is needed to achieve sufficient statistical power and accurately assess the genetic architecture in Aswan.
3. Population-Specific Findings: The population in Aswan is a rich blend of diverse genetic, ethnic, and cultural backgrounds, including Nubians, Arabs, Upper Egyptians (Sa’idis), and descendants of Sudanese people, making genetic studies more challenging in this region.
4. The study lacks functional analysis to connect SNPs with disease mechanisms, leaving the biological significance speculative.
5. Environmental factors, lifestyle, treatment regimens, and coexisting conditions complicate the isolation of genetic contributions from PTPN22 and PADI4 polymorphisms.
6. Genetic variation and SNPs: The variability in results across different populations may be attributed to genetic differences, particularly single nucleotide polymorphisms (SNPs).

## Conclusion

While PTPN22 and PADI4 were not significantly associated with RA risk in the Aswan population, their correlation with disease activity markers such as ACPA suggests a potential diagnostic role. Our findings indicate that PTPN22 is significantly linked to RF and ACPA levels, respectively, highlighting its potential as a diagnostic marker. However, its role in disease progression remains uncertain. PADI4, in particular, shows promise as a negative diagnostic marker because of its high specificity. However, further studies with larger sample sizes and functional analyses are needed to explore the exact role of these genes in RA susceptibility and disease progression.

## Data Availability

De-identified individual-level data supporting our findings, can be requested from Dr. Khaled Abdelsalam Abdien Abdelgalil at the Department of Rheumatology, Aswan University, Egypt (khaled.abdelsalam@med.aswu.edu.eg). Access requires approval from the Aswan University Faculty of Medicine Research Ethics Committee (IRB Ref No: Asw.Uni./678/10/22) and is available under a data-sharing agreement for non-commercial research for five years post-publication.

## Abbreviations

RA: Rheumatoid Arthritis
PTPN22: Protein Tyrosine Phosphatase Non-Receptor Type 22 (gene)
PADI4: Peptidyl Arginine Deiminase 4 (gene)
RF: Rheumatoid Factor
ACPA: Anti-Citrullinated Protein Antibody
DAS28: Disease Activity Score in 28 Joints
ESR: Erythrocyte Sedimentation Rate
CRP: C-Reactive Protein
PGA: Patient Global Assessment
SNP: Single Nucleotide Polymorphism
HLA: Human Leukocyte Antigen
ELISA: Enzyme-Linked Immunosorbent Assay
PCR: Polymerase Chain Reaction
AUC: Area Under the Curve (ROC analysis)
ROC: Receiver Operating Characteristic
OR: Odds Ratio
CI: Confidence Interval
ACR: American College of Rheumatology
EULAR: European Alliance of Associations for Rheumatology
HAQ: Health Assessment Questionnaire
IQR: Inter-quartile Range
SD: Standard Deviation
