# Correction: Associations of PTPN22 and PADI4 polymorphisms with rheumatoid arthritis in ASWAN

**DOI:** 10.1186/s41927-025-00578-9

**Published:** 2025-10-13

**Authors:** Khaled A. A. Abdelgalil, Nihal Fathi, Fatma H. El Nouby, Nour A. Mohammed, Loay I. Aglan

**Affiliations:** 1 Department of Rheumatology, Rehabilitation & Physical Medicine, Faculty of Medicine, Aswan University, Aswan, Egypt; 2 Department of Rheumatology, Rehabilitation & Physical Medicine, Faculty of Medicine, Assiut University, Assiut, Egypt; 3 Department of Clinical Pathology, Faculty of Medicine, Aswan University, Aswan, Egypt

**Correction: BMC Rheumatol 9**,** 105 (2025)**

10.1186/s41927-025-00566-z

Following publication of the original article [1], the name of corresponding author “Khaled A. A. Abdelgalil” has been corrected.

The original article [1] has been updated.
